# Atherogenic Index and High-Density Lipoprotein Cholesterol as Cardiovascular Risk Determinants in Rheumatoid Arthritis: The Impact of Therapy with Biologicals

**DOI:** 10.1155/2012/785946

**Published:** 2012-09-06

**Authors:** Calin D. Popa, Elke Arts, Jaap Fransen, Piet L. C. M. van Riel

**Affiliations:** Department of Rheumatology, Radboud University Nijmegen Medical Centre, P.O. Box 9101, 6500 HB Nijmegen, The Netherlands

## Abstract

Cardiovascular (CV) diseases are a serious concern in rheumatoid arthritis (RA), accounting for approximately one-third to one-half of all RA-related deaths. Besides the attempts to identify new risk factors, the proper management of traditional CV risk factors such as dyslipidemia should become a priority in the periodic evaluation of every RA patient. Atherogenic index has been suggested to be less susceptible to disease activity variation during large periods of time, making him more attractive to be used in CV risk prediction in this group of patients as compared to individual lipids concentrations. Nevertheless, inflammation may negatively impact HDL antiatherogenic properties, suggesting that HDL function assessment is of particular importance when predicting CV risk in these patients. A tight control of inflammation becomes therefore crucial for a successful CV risk management. The present paper debates these hypotheses focusing on the effects of therapy with biologicals on the above mentioned parameters.

## 1. Introduction

Cardiovascular (CV) diseases are a serious concern in patients with chronic inflammatory diseases. For patients with rheumatoid arthritis (RA), it represents the leading cause of death, accounting for approximately one third to one half of all RA-related deaths [1, 2]. In order to decrease this incidence, risk factors need to be identified in the first place. Intriguingly, previous studies have suggested that the augmented CV burden found in RA patients seems not to be fully explained by traditional CV risk factors, such as dyslipidemia, hypertension, smoking, and physical inactivity [3]. Consequently, factors leading or deriving from the chronic inflammation have been suggested to be responsible for the augmented risk [4–6]. Until nowadays, however, no such factor is proved to solidly confirm this hypothesis.

Recently, several studies have suggested that it might have been enough room to improve the cardiovascular profile of RA patients only by focusing on the traditional risk factors. Impaired during the periods of active disease, physical activity could be importantly improved by a better disease control as suggested in the recent international guidelines, consequently improving CV profile [7]. Using different methods to assess the risk of developing CVD, Toms et al. have recently reported that between 2% and 25% of RA patients who should receive a lipid-lowering drug (statin) according to their calculated risk do not actually use this medication [8]. The percentages may even increase from 7% to 30% if the 1.5 multiplier factor is applied as recently recommended [9]. Despite its limitations, the study emphasizes the possibility of suboptimal therapy of traditional risk factors in RA patients, providing a solid alternative to improve CV pattern in RA. Finally, inflammation may alter traditional CV risk factors including lipids pattern, both at the concentration and composition level [10, 11]. This observation has recently led to the concept of “smaller slice of a bigger pie,” which emphasizes that due to the presence of chronic inflammation, the relative contribution of these factors to the overall CV risk in RA is different than in the general population. All these data suggest that despite the progresses made in the past years, traditional CV risk factors such as dyslipidemia are not yet entirely understood and appropriately managed in patients with RA.

Traditionally, the atherogenic lipid profile is made up of increased TC, LDL, TG, and decreased HDL. In chronic inflammatory diseases such as RA, however, different concentrations of lipids can be found throughout different stages of the disease: increased TC and LDL in the years prior to disease onset, reduced levels of TC and HDL-C during early active disease, and different patterns in established RA [12, 13]. Hence, due to the variable degree of chronic inflammation, the individual lipid concentrations may frequently fluctuate during the course of disease making the impact of such changes on CV risk less clear. Nevertheless, the different cholesterol fractions seem to fluctuate together in the same direction. In line with this, recent studies have suggested that the atherogenic index (AI—the ratio TC : HDL) is less susceptible to disease activity fluctuations in RA. Therefore, one can hypothesize that AI may be more appropriate to be used to assess the relative contribution of lipids to the CV risk in RA patients than individual cholesterol fractions measurements. Finally, inflammation may not only modulate the levels but also the composition of lipoproteins. In line with this, our group and others have shown that HDL becomes less antiatherogenic in RA patients, and this is associated with inflammatory status [10, 11]. Therefore, we suggest that in chronic inflammatory conditions, HDL anti-atherogenic properties (i.e., antioxidant, cholesterol reverse transport) may prove to be a valuable alternative marker to predict the development of atherosclerosis and CV burden in RA patients.

Recent recommendations for the treatment of RA propose a tight control of disease activity to achieve rapid remission in the early disease stage. Controlling the inflammatory process is likely to favorably impact CV risk. In line with this, new therapeutic strategies have been recently elaborated, encouraging the use of aggressive antirheumatics, including biologicals, earlier in the course of disease [7]. The consequence will be that an increasing number of RA patients will be treated in the future with these drugs. Appropriate knowledge about their effects on cardiovascular risk factors, including lipid pattern, would therefore be of great importance. Several previous publications have addressed the effects of biologicals on the lipid profile, concentrating on individual lipid levels/changes. However, important questions regarding the overall atherogenic capacity of the lipid profile and the subsequent impact on the cardiovascular risk remain largely unanswered. The present paper focuses on the relation between the therapy with biologicals and atherogenic index as a more suitable parameter in RA to address CV risk in this population. In addition, data on HDL function in the same context will be discussed.

## 2. Methods

### 2.1. Literature Search and Study Selection

We conducted a literature search in Medline via PubMed for articles published up to May 2012. The MeSH terms used were anti-TNF, infliximab, adalimumab, etanercept, tocilizumab, rituximab, and rheumatoid arthritis (MeSH). These were combined with cholesterol (MeSH), lipids, HDL, and atherogenic index. Articles were selected if they met all of the following criteria: (a) clinical trial or observational study that included ≥ 10 patients with rheumatoid arthritis (except for rituximab studies), (b) treatment with infliximab, adalimumab, etanercept, tocilizumab, or rituximab, and (c) values of total cholesterol (TC), HDL, and atherogenic ratio's taken before and after treatment. The search was further restricted to English language full-text articles. Studies were manually selected by two authors (CP, EA) by screening the title, keywords, and abstract, using the eligibility criteria. If possibly eligible, full-text articles were retrieved and judged using the eligibility criteria. The inclusion of articles was determined by consensus.

### 2.2. Data Presentation

Due to the heterogeneity of study populations, type of treatment, dosages, follow-up time, outcome measures, and statistical analysis, a meta-analysis was not performed. Hence, a narrative summary of the results is provided. The primary summary measure used to compare results was the difference in AI for short-term studies (<6 months) and long-term studies (>6 months). Results regarding anti-TNF*α*, anti-IL-6R, and anti-CD20 therapy are discussed. No additional quality assessments were performed. Sample size, differences in type of treatment and dosages, and study duration were taken into consideration when comparing results.

## 3. Results and Discussion

**Figure 1:**
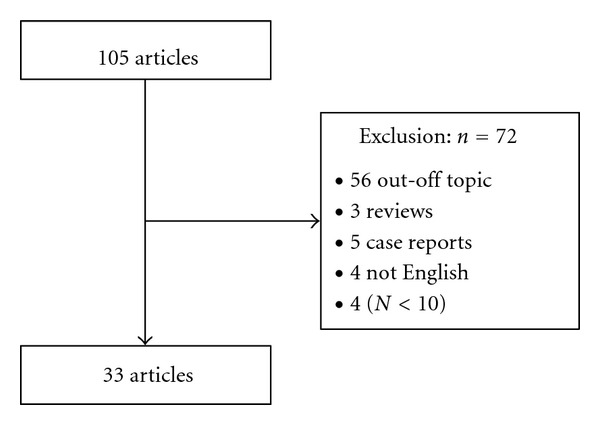
Flowchart.

In total, there were 105 records identified. Of them, 4 were excluded because they were not written in English, 5 were case reports, 56 were off topic, 3 were themselves reviews, and 4 studies investigated less than ten RA patients (see inclusion criteria). At the end of the selection procedure, 33 full-text articles met the eligibility criteria and were considered for this paper (Figure 1). Of the 33 studies, the vast majority concerned anti-TNF users, usually infliximab, adalimumab, and etanercept [11, 14–32], 8 studies concerned tocilizumab (including three randomized clinical trials) [21, 33–39], and 5 studies investigated rituximab effects on lipids pattern [14, 40–43]. Data on other biologicals, including abatacept, anakinra, golimumab, or certolizumab have not been addressed here due to their very limited and preliminary character. 

### 3.1. Anti-TNF Agents

TNF-*α* is a proinflammatory cytokine which plays a pivotal role in both RA and atherosclerosis pathogenesis. A beneficial effect of anti-TNF treatment on CV morbidity and mortality in RA has been demonstrated [44]. Many studies have investigated the effects of anti-TNF medication on the lipid profile, yet the majority of studies comprise small groups of patients with a short followup. This paper will further focus on studies concerning infliximab, adalimumab, and etanercept. As previously mentioned, it will separately address the short and long-term effects, respectively, for all three drugs taken together. Finally, the effects on HDL function will be summarized.

#### 3.1.1. Short-Term Studies

Short-term studies demonstrate primarily significant antiatherogenic changes, particularly in TC and HDL levels, whereas TG and LDL concentrations often remain unchanged. Interestingly and of importance for our present paper, changes in the atherogenic index (TC : HDL) and other ratios (LDL : HDL, ApoB : ApoA-1) have also been noticed. Our group found a significant decrease of approximately 8% in both LDL : HDL and the TC : HDL ratio after two weeks of treatment with adalimumab in a group of 33 RA patients as compared to placebo [24]. Our results have been further confirmed by a recent study in 50 RA patients receiving adalimumab: AI baseline—16 weeks was 3.33 (0.93) versus 3.15 (0.85), *P* = 0.034 [32]. A significant decrease in the apoB : apoA-1 ratio has been also reported (*P* = 0.014). A trend towards a more pronounced effect on HDL in the responders group has been noticed together with an association with disease activity changes (*r* = −0.31, *P* = 0.03). Similar results have been reported by Jamnitski et al., who found a significant decrease in the ApoB : ApoA-1 ratio over a period of 3 months [19] in 292 RA patients receiving TNF blockade. Interestingly, this change has been found only in good and moderate EULAR responders. Nevertheless, some further studies reported opposite results (Table 1). Following 45 RA patients treated with infliximab during a period of almost 6 months, our group reported a significant increase in the TC : HDL ratio [11] at the end of this period. These findings were supported by Dahlqvist et al. [17], who reported an increase of 8% and 9% in the LDL : HDL and TC : HDL ratio, over the same time period in 52 RA patients treated with infliximab. Other studies did not indicate any change in the atherogenic index or other ratios within a period of 3 or 6 months of anti-TNF therapy [14, 16, 18, 21, 25, 26, 28, 30, 31], although individual lipid levels were often found to increase in the initial months of treatment [25, 26, 30, 31]. Studying 56 patients with RA receiving infliximab for 30 weeks, Allanore et al. found no changes in the atherogenic index despite a significant stable increase of HDL and TC. They also noticed no relations between response to therapy and lipid pattern modifications [15]. Similar findings have been reported by Seriolo et al. in 34 consecutive RA patients treated with various TNF blockers (*n* = 16 for etanercept, *n* = 14 for infliximab, and *n* = 4 for adalimumab) for 24 weeks [26]. The authors reported however on a relation between changes in HDL and disease activity (DAS28) by the end of the study (*r* = −0.52, *P* < 0.01), without making any reference to response rate. These findings are in line with those from a previous study, indicating a correlation between the decrease in disease activity and the increase in HDL 6 weeks after therapy with infliximab has been initiated [31]. This association remained after adjusting for changes in prednisone dose, age, gender, and disease duration. Although the mean atherogenic index did not change, changes in DAS28 were significantly associated with changes in the atherogenic index in the period 0 to 2 weeks. However, this association disappeared when the whole study period (6 weeks) has been considered.

A few more studies should be mentioned, which did investigate the effects of TNF blockade on lipids pattern in RA patients, however, without entirely fulfilling our inclusion criteria. Several investigators pulled together data from patients with RA and other inflammatory conditions such as ankylosing spondylitis [20]. In this setting, they found no changes in AI after 6 months of therapy with infliximab. Other studies provided data only on individual lipids without atherogenic index or other ratios [22, 27, 29]. Finally, in an elegant study, Gonzalez-Juanatey et al. investigated endothelial function and atherogenic index in a small group (*N* = 8) of RA patients who failed on infliximab and were now treated with adalimumab. Besides rapid improvement of endothelial function, a significant decrease of the atherogenic index was observed at week 2 (3.30 ± 0.55) and at week 12 (3.28 ± 0.48) when compared with baseline atherogenic index result (3.52 ± 0.50) (*P* value for both comparisons = 0.012). This was associated with a decrease in disease activity and inflammation status [45].

The apparent heterogeneity of these results may be due to several factors. Firstly, it mostly concerns small-group studies enrolling RA patients from diverse countries with a distinctive health care system and lifestyle habits, including physical activity (biking for the Dutch population) [11, 23–25, 31, 32] and alimentation (fish-reach diet in Northern Europe, Mediterranean diet in the Southern Europe) [14, 15, 17, 18, 20, 26, 28]. Secondly, a difference between the anti-TNF agents may be present, leading to a more pro-atherogenic profile in the case of infliximab [11, 17], with milder effects for adalimumab and etanercept [19, 24, 32]. Thirdly, gender may also contribute to this heterogeneity, our group reporting a more pronounced effect on lipid pattern in male RA patients. Accordingly, total cholesterol and HDL increased more markedly 6 months after starting infliximab (*P* < 0.04), translating into a tendency to increase of the atherogenic index [25]. Finally, the response rate and the degree of response to anti-TNF therapy is likely to impact the changes in lipid profile. Though several studies have addressed the association between changes in disease activity or inflammatory status and changes in lipids concentrations, only a few investigated the association between the latter and response according to established criteria (EULAR/ACR) [19, 25, 32]. These studies suggest that the atherogenic index tends to increase more in nonresponders as compared to responders [25], or to decrease only in responders [19, 32].

#### 3.1.2. Long-Term Studies

During the first year of treatment with anti-TNF agents, lipid concentrations tend to increase, with some reporting a return to baseline levels after an initial increase [23]. Despite a constant dosage of the anti-TNF drug, changes in AI reported by short-term studies are often not sustained over longer periods of time. Using etanercept in a group of 292 RA patients, Jamnitski et al. found a more pronounced decrease of apoB : apoA-1 ratio 4 months after therapy has been initiated as compared to one year time-point, whereas TC : HDL ratio remained similar throughout study period [19]. The authors have also performed an analysis in patients who responded and patients who did not respond to the therapy according to the EULAR response criteria. There was a trend towards a lower AI both 4 months as well as one year after starting etanercept in the responders subgroup as compared with the nonresponders, reaching significance in the case of apoB : apoA-1 ratio (*P* = 0.005). Wijbrandts et al. also reported an improvement of the atherogenic index 52 weeks after adalimumab has been started in a group of 44 RA patients [32], with apoB : apoA-1 ratio decreasing with 7% (*P* = 0.05) and TC : HDL ratio with 4% (*P* = 0.27). Of note, both ratios reached statistical significance 16 weeks after starting adalimumab (*P* = 0.014 and *P* = 0.034, resp.) [32]. In contrast, in a case-control study of 52 established RA patients and 70 early RA patients, Dahlqvist et al. reported that the LDL : HDL and TC : HDL ratios significantly worsened one year and even two years after infliximab was started: 9.2% and 10.4%, respectively [17]. In line with this, our group found a significant increase in the TC : HDL ratio in a group of 55 RA patients treated with infliximab: 9% after 6 months (*P* = 0.02) and 4% after 12 months (*P* = 0.05) [25]. In the same study, LDL : HDL ratio did not significantly changed over time. Peters et al. found no change in the apoB : apoA-1 ratio and TC : HDL ratio, respectively, in a group of 80 RA patients treated with infliximab for a period of 48 weeks [23]. Interestingly, they observed that changes in prednisone dose were related to changes in HDL and TC, with a relatively greater impact on HDL, resulting in a inverse association between prednisone dose and atherogenic index (TC : HDL and apoB : apoA-1) [23]. Finally, in a large study involving different anti-TNF agents (infliximab, adalimumab, and etanercept), Ajeganova et al. found no changes in apoB : apoA-1 ratio in all three subgroups according to the drug, 12 months after therapy has been initiated [14]. Similar results have been previously reported by Engvall et al., who observed no change in apoB : apoA-1 ratio between 3 months and 2 years of followup [18]. Both studies report no data on TC : HDL index.

Despite apparent discrepancy, some trends may be depicted when analyzing these long-term effects of anti-TNF drugs on lipids in patients with RA. These trends become clearer when focusing on atherogenic index, which demonstrates therefore to be superior to individual lipid concentrations in this respect (Table 2). Therapy with etanercept or adalimumab seems to have a positive impact on atherogenic index, although this improvement does not always reach statistical significance [19, 32]. In contrast, the use of infliximab may worsen lipid ratios on the long term [17, 25], though some report a neutral effect [23]. Nevertheless, a rapid and sustained control of disease activity as in the case of responders would be associated with better ratios as compared to non-responders, even in those patients treated with infliximab [23]. Alternatively, the concomitant use of prednisone may influence atherogenic index. Given the prognostic value of these ratio for future CV events, it is likely that these changes are clinically relevant and may contribute to the decreased incidence of myocardial infarction and other CV events observed with anti-TNF*α* treatment in RA.

#### 3.1.3. Anti-TNF Therapy and HDL Function

The link between HDL and cardiovascular disease risk is far more complex than originally thought. This may be explained by the inherent heterogeneity of HDL particles in terms of composition, structure, and biological function. Emerging evidence suggests that for instance small dense protein-rich HDL3 particles are less capable of protecting LDL against oxidative modification [46]. This has led some to propose that the functionality of HDL may be as relevant as plasma levels of HDL to cardiovascular risk assessment [47, 48]. In the same context, a number of studies have demonstrated that inflammation is able to negatively impact the anti-atherogenic properties of HDL [49]. The issue becomes of interest thus in the case of patients suffering from chronic inflammatory diseases, such as RA.

In a study on 48 RA patients, which also included patients with SLE and healthy controls, McMahon et al. demonstrated for the first time the presence of a pro-inflammatory HDL in this group of patients [10]. About 20% of RA patients were likely to have such an HDL, as compared to 4% of healthy controls. HDL function tended to correlate with ox-LDL concentrations (*r* = 0.355). Inflammatory markers and prednisone dosage have been shown to be associated with a proinflammatory HDL. Interestingly, the authors found no association between HDL function (proinflammatory) and HDL concentrations, an observation which has been recently confirmed by an elegant study in the general population [47]. Statins may reverse the pro-inflammatory HDL in a small group of RA patients during a period of 12 weeks [50]. This improvement was not entirely associated with a decrease in inflammatory state. It was further indicated that the pro-inflammatory function of HDL in RA might be due to a different composition as compared with anti-inflammatory HDL (51), including a lower LCAT activity and higher MPO activity. Nevertheless, the study does not provide sufficient evidence to support the standard use of statins in patients with RA.

Our group has investigated for the first time the effects of anti-TNF therapy on HDL antiatherogenic function. We found that infliximab is able to improve HDL antioxidative capacity, an effect that was sustained 6 months after anti-TNF therapy has been initiated [11]. It is still unclear how stable these effects are further in the course of therapy and whether they are solely due to TNF blockade or more likely to reflect the overall inflammatory suppression achieved in these patients. Recently, we observed that HDL subfractions are modified in RA patients, especially in women [51], reinforcing again the importance and in the same time the complexity of HDL status in these patients with respect to their cardiovascular risk. Whether anti-TNF drugs are able to restore this detrimental HDL profile remains a subject for further investigations.

### 3.2. Anti-IL6 Agents

Interleukin (IL6)- is another cytokine that plays a key role in the pathogenesis of chronic inflammatory diseases. Recently, the therapeutic blockade of its receptor proved to efficiently suppress disease activity in patients with RA [33–35, 37, 39]. Owing to the increased cardiovascular risk and anti-TNF experience, trials investigating the effects of tocilizumab (TCZ), the IL-6 receptor (IL-6R) antagonist in patients with RA have included for the first time the impact of the therapy on the lipid pattern as part of the safety analysis of the drug. An increase of individual lipid concentrations has been constantly reported with TCZ [37, 38]. Nevertheless, detailed results regarding the effect of treatment on the atherogenic index could not be derived from all of the studies (Table 3). Maini et al. reported that lipids levels increased initially and then stabilized and did not continue to increase during the treatment period, which is comparable to the effects reported in anti-TNF studies. Importantly, the mean atherogenic index remained largely unchanged and was reduced to below its initial level by the 20-week follow-up visit in the groups receiving 8 mg/kg of TCZ [37]. In another trial by Emery et al., 20-week therapy with TCZ resulted in higher rate of more than 30% increase in LDL/HDL ratio in patients receiving the drug as compared to controls: 22.2% (TCZ 8 mg/kg), 19.1% (TCZ 4 mg/kg), and 10,1% (controls), respectively [33]. In contrast, comparable proportions of patients had greater than 30% increase in the apoB/apoA ratio: 11.6% (TCZ 8 mg/kg), 9.4% (TCZ 4 mg/kg), and 9.7% (controls), respectively. No acute cardiovascular event has been reported during the study period. In the OPTION study comparing two TCZ regimens with placebo, Smolen et al. report similar results [39]. Increases in the ratio of total cholesterol to HDL of more than 30% above baseline were observed in 17% of patients treated with TCZ 8 mg/kg, 8% of patients receiving TCZ 4 mg/kg and 5% in the placebo group. Comparable apoB/apoA ratio between the groups have been reported however. One last trial adds to strengthen the previous presented data (TOWARD study) [34]. It compared patients receiving TCZ 8mg/kg and a DMARD with patients receiving a DMARD and placebo. The authors indicate increases of more than 30% in the TC/HDL ratio in 12% and 7% of patients in the TCZ and control group, respectively, and increases of more than 30% in the LDL/HDL ratio in 20% and 12% of patients, respectively. Again, no significant changes in the apoB/apoA ratio have been noticed in both groups. Finally, Jones et al. compared the monotherapy with TCZ and methotrexate in a group of 673 RA patients (AMBITION study) [35]. They report no data on atherogenic index during the 24 weeks of therapy. It was however noted that TCZ is more prone to disturb lipid pattern as compared to methotrexate and leads to LDL and triglycerides elevations. In an observational study, Kawashiri et al. noticed no changes in the ApoB/ApoA-1 and TC/HDL ratio despite an increase of individual lipids in a small group of RA patients treated with TCZ for 12 weeks [36]. Similar findings have been reported by Kume et al., who found no changes in TC/HDL ratio 24 weeks after starting tocilizumab in 22 RA patients, despite sustained increase of both TC and HDL alone [21]. Interestingly, the authors noticed that the increase in TC in the TCZ group has been higher than in the patients receiving adalimumab or etanercept, reaching statistical significance (TCZ versus ETN *P* = 0.024, TCZ versus ADA *P* = 0.032). Although the first of its kind by directly comparing three different biologicals with respect to endothelial dysfunction and lipid pattern, the results of the study should be interpreted with caution given the relative low number of patients enrolled in each group (approximately 20).

Overall, the present experience with tocilizumab appears to suggest a certain detrimental effect on lipids pattern, translated into a higher percentage of patients with a significant increase in the atherogenic index—TC/HDL and LDL/HDL [33, 34, 39], whereas apoB/apoA-1 ratio remains stable throughout the therapy [33, 36, 39]. These lipid modifications led in several cases to the start of therapy with lipid-lowering agents. It is still unclear if long-term treatment with TCZ would reverse these detrimental effects and achieve sustained improvements in AI. To our knowledge, no studies have investigated the effect of TCZ on the HDL cholesterol function. Given the emerging importance of this factor in CVD risk assessment, future studies on this issue are warranted.

### 3.3. Rituximab

Up to date, there are few studies investigating the effects of newer biologicals on lipids pattern in RA patients. Rituximab, a B-cell depletion drug, targeting the CD20 positive B lymphocytes, has been so far scarcely investigated for its effects on atherogenic index and HDL composition, as compared to anti-TNF drugs. In a small group of RA patients, Gonzalez-Juanatey et al. investigated for the first time the effects of rituximab on lipid parameters [40]. The authors have found a slight, nonsignificant increase in HDL levels both 2 weeks (56 ± 11 mg/dl) and 6 months (57 ± 15 mg/dl) compared to baseline (52 ± 11 mg/dl), whereas total cholesterol increased only 2 weeks after starting rituximab (211 ± 42 mg/dl versus 191 ± 37 mg/dl). No direct information on atherogenic index has been provided. In another study, Kerekes et al. found an increase in HDL levels with 14.3%, 33.1%, and 35.4% as compared to baseline, at 2, 6, and 16 weeks, respectively, after rituximab has been initiated [41]. At sixteen-week time-point, the difference reached significance (*P* = 0.035). Interestingly, total cholesterol tended to decrease without significance, throughout study period. This may suggest a decrease in the atherogenic index. The results are likely in line with the previous ones, yet the limited number of patients investigated (*n* = 5) makes their interpretation difficult. The first larger study on this issue comes from Ajeganova et al. [14]. The Swedish group investigated the effects of various biologicals on lipids pattern in 215 RA patients receiving therapy with various biologicals, focusing on apolipoproteins (apoA and apoB) and their ratio. The investigators found that in the rituximab-treated group (*n* = 53) apoA-1 levels increased throughout the study with 0.09 ± 0.32 g/L (*P* = 0.022, followup of 6 months) and 0.09 ± 0.32 g/L (*P* = 0.06, followup of 12 months), respectively. The ratio apoB/apoA-1 remained relatively stable and did not change significantly over the study period. The TC, HDL, and their ratio (AI) have been not assessed. Interestingly, the authors found no associations between apoB/apoA-1 ratios and markers of disease activity, therefore sustaining our hypothesis that ratios are less susceptible to changes in disease activity and thus likely more proper to predict CV risk in these patients. Finally, two more studies should be mentioned, which further investigated the interplay between rituximab and lipids in RA patients by assessing the effects of this drug on HDL antiatherogenic function [42, 43]. In the first one, 49 RA patients have been followed 6 months after receiving rituximab [43]. As previously suggested, rituximab modestly increased HDL and apoA-1 levels and significantly improved atherogenic index (*P* < 0.05). A subanalysis revealed that these changes were only present in the subgroup of responders. There is no association found with the use of prednisone. HDL composition changed upon rituximab therapy, becoming depleted in SAA-1 in patients who have demonstrated a good response to the therapy, rendering the molecule to be anti-atherogenic. This observation further substantiates the importance of HDL function assessment in patients with RA and other chronic inflammatory conditions in order to get a proper picture of their CV risk. In the second study, Mathieu et al. presented data on 33 RA patients treated with rituximab [42]. Atherogenic index remained stable, although TC significantly increased both 6 and 12 months after rituximab (*P* < 0.001). The study enrolled RA patients with longer disease duration (mean 17.6 years) who have already fallen on two anti-TNF drugs.

## 4. Concluding Remarks

The available literature shows that anti-TNF drugs, IL-6R antagonists, and anti-CD20 antibodies are able to modulate the lipid profile in RA. Interestingly, when considering their effects on the atherogenic index and other lipoproteins ratio, it becomes evident that changes in individual lipid levels often do not translate into a change in AI, or are not sustained long enough to significantly affect the atherogenic index. Therapy with etanercept, adalimumab, or rituximab seems to have a positive impact on atherogenic index, although this improvement does not always reach statistical significance and sometimes an initial gain is lost over time. In contrast, the use of infliximab may worsen lipid ratios on the long term, though some report a neutral effect. Similarly, tocilizumab is likely to worsen lipid ratios in the first months after therapy has been initiated, while the longer-term effects remain still unknown. Nevertheless, controlling disease activity and achieving remission seem to beneficially impact the lipid pattern, as suggested by the positive effects seen in responders. Finally, the form and function of HDL appear to be compliant to changes in inflammation. Treatment with anti-TNF agents and rituximab results in improvements of the HDL antiatherogenic capacity. It is unclear whether these changes progress over time and to what extent they decrease the CV risk. No data on the effects of tocilizumab on HDL function are available.

The interpretation of our conclusions should not be without caution. It is still unclear to what extent these changes actually lead to a change in the CV risk. Moreover, some suggest that even if changes occur, they might have a milder impact degree on CV risk as compared to the general population [52]. The follow-up period of these studies is often too short to include CV events. Sometimes possible confounding variables are not accounted properly for the effect on lipids, which is not a primary outcome for instance in the majority of tocilizumab studies.

In conclusion, we suggest that atherogenic index and HDL function are more suitable parameters of lipid profile as determinants of CV risk in patients with RA, and perhaps for other chronic inflammatory diseases including lupus, psoriatic arthritis, and ankylosing spondylitis. The effects of biologicals on these parameters depend on the response rate, concomitant prednisone use, duration of therapy, and the biological self. If CV risk management will become an integrated part of RA therapeutic strategies, and given the increasing importance of personalized medicine, the choice of biological might be done in the future also in accordance with its own CV risk profile, where its effect on lipids pattern will become of crucial value, as presented above. Future studies with clinical CV endpoints would have to address the value of monitoring AI and HDL function during therapy with biologicals in order to establish their real impact on CV risk in these patients.

## Figures and Tables

**Table 1:** Short-term effects of anti-TNF drugs on atherogenic index and other ratios.

Study	Drug	Number of patients	Duration	Effect	
				AI	Other ratios
Popa et al. [24]	ADA	33	2 wk	L	LDL : HDL
Wijbrandts et al. [32]	ADA	50	16 wk	L	apoB : apoA-1
Gonzalez-Juanatey et al. [45]	ADA	8	12 wk	L	—
Kume et al. [21]	ADA/ETN	42	24 wk	n	—
Seriolo et al. [26]	ADA/ETN/IFX	34	24 wk	n	—
Soubrier et al. [28]	ADA/ETN/IFX	29	14 wk	n	apoB : apoA-1
Jamnitski et al. [19]	ETN	292	16 wk	L	apoB : apoA-1
Allanore et al. [15]	IFX	56	30 wk	n	LDL : HDL
Popa et al. [11]	IFX	45	24 wk	H	—
Dahlqvist et al. [17]	IFX	52	24 wk	H	—
Popa et al. [25]	IFX	55	24 wk	H	—
Tam et al. [30]	IFX	19	14 wk	n	LDL : HDL
Vis et al. [31]	IFX	69	6 wk	n	—
Engvall et al. [18]	IFX	40	14 wk	—	apoB : apoA-1
Ajeganova et al. [14]	ADA/ETN/IFX	162	24 wk	—	apoB : apoA-1
Curtis et al. [16]	not specified	289	8 wk	L	—

ETN: etanercept, ADA: adalimumab, IFX: infliximab, AI: atherogenic index, wk: weeks, L: lower, n: neutral, H: higher.

**Table 2:** Long-term effects of anti-TNF drugs on atherogenic index and other ratios.

Study	Drug	Number of patients	Duration	Effect	
				AI	Other ratios
Jamnitski et al. [19]	ETN	292	1 year	n	apoB : apoA-1
Wijbrandts et al. [32]	ADA	50	1 year	n	apoB : apoA-1
Dahlqvist et al. [17]	IFX	52	2 years	H	—
Popa et al. [25]	IFX	55	1 year	H	LDL : HDL
Peters et al. [23]	IFX	80	1 year	n	apoB : apoA-1
Ajeganova et al. [14]	ETN/ADA/IFX	162	1 year	—	apoB : apoA-1
Engvall et al. [18]	IFX	18	2 years	—	apoB : apoA-1

ETN: etanercept, ADA: adalimumab, IFX: infliximab, AI: atherogenic index, n: neutral, H: higher.

**Table 3:** Effects of tocilizumab on atherogenic index and other lipid ratios.

Atherogenic index	Study (ref), patients (*N*), and lipid ratio's
Higher	Emery et al. [33] (*N* = 338) LDL/HDL; Genovese et al. [34] (*N* = 803) TC/HDL, LDL/HDL; Smolen et al. [39] (*N* = 418) TC/HDL

Neutral	Kume et al. [21] (*N* = 22) TC/HDL; Kawashiri et al. [36] (*N* = 19) TC/HDL, apoB/apoA-1; Maini et al. [37] (*N* > 50) TC/HDL;

N.A.	Jones et al. [35]; Schultz et al. [38]

N.A.: not assessed.
